# Construction of a Genome-Scale Kinetic Model of Mycobacterium Tuberculosis Using Generic Rate Equations

**DOI:** 10.3390/metabo2030382

**Published:** 2012-07-03

**Authors:** Delali A. Adiamah, Jean-Marc Schwartz

**Affiliations:** Manchester Institute of Biotechnology, Faculty of Life Sciences, University of Manchester, 131 Princess Street, Manchester M1 7DN, UK

**Keywords:** tuberculosis, metabolism, kinetic model, systems biology

## Abstract

The study of biological systems at the genome scale helps us understand fundamental biological processes that govern the activity of living organisms and regulate their interactions with the environment. Genome-scale metabolic models are usually analysed using constraint-based methods, since detailed rate equations and kinetic parameters are often missing. However, constraint-based analysis is limited in capturing the dynamics of cellular processes. In this paper, we present an approach to build a genome-scale kinetic model of *Mycobacterium tuberculosis* metabolism using generic rate equations. *M. tuberculosis* causes tuberculosis which remains one of the largest killer infectious diseases. Using a genetic algorithm, we estimated kinetic parameters for a genome-scale metabolic model of *M. tuberculosis* based on flux distributions derived from Flux Balance Analysis. Our results show that an excellent agreement with flux values is obtained under several growth conditions, although kinetic parameters may vary in different conditions. Parameter variability analysis indicates that a high degree of redundancy remains present in model parameters, which suggests that the integration of other types of high-throughput datasets will enable the development of better constrained models accounting for a variety of *in vivo* phenotypes.

## 1. Introduction

Genome-scale metabolic models are essential to bridge the gap between metabolic phenotypes and genome-derived biochemical information, as they provide a platform for the interpretation of experimental data related to metabolic states and enable the *in silico* experimentation of cell metabolism. The annotation and sequencing of genomes has made it possible to reconstruct genome-scale metabolic networks for a growing number of organisms [1]. Using constraint-based methods and *in silico* simulation, the phenotypic functions of metabolic systems can be analysed under various environmental or physico-chemical conditions [2]. Applications of these computational methods to bacterial metabolic models have increased our understanding of bacterial evolution and metabolism [3]. Genome-scale models additionally allow for the integration of various types of high-throughput data. For example, the integration of regulatory interactions with metabolic networks has been successfully used to analyse phenotypes from gene-deletion studies and phenotypic arrays [4]. Other types of data, such as metabolomics and proteomics, have also been integrated with constraint-based methods [5].

The availability of genome-scale metabolic networks has accelerated the development of methods to analyse system-wide metabolic properties. A fundamental aim of systems biology is to predict cellular behaviours *in silico* by examining the dynamics of cellular processes [6]. As a result, it is necessary to go beyond static constraint-based models and build kinetic models where systems can be perturbed [7]. However, it is time-consuming and costly to experimentally measure all metabolite concentrations, reaction fluxes and kinetic parameters at the genome scale. This has led to recent efforts to providing methods to build kinetic models using other approaches, such as linlog kinetics [8,9], generic equations [10], parameter balancing [11] and convenience kinetics [12].

Reverse engineering is often used in systems biology to reconstruct biological interactions and constrain kinetic parameter values from experimental data [13]. It is often unlikely to have access to comprehensive datasets comprising all metabolic, genomic and proteomic data needed to fully constrain kinetic parameter values, and as such, simulated or calculated data may be used as a substitute. Flux Balance Analysis (FBA), which enables the calculation of an optimal flux distribution using linear programming, has proved an efficient method to represent metabolic phenotypes under various experimental conditions, with successful prediction rates found to be approximately 60 and 86% for *H. pylori* and *E. coli* respectively in gene deletion studies [14]. As kinetic parameters are not required for FBA, a flux distribution can be calculated in a genome-scale metabolic model when only the network stoichiometry and flux constraints are known. In Lubitz *et al*. [11] the authors used a technique known as ‘parameter balancing’, which is based on Bayesian parameter estimation, to estimate kinetic parameters of metabolic reactions. This method was validated on the phosphofructokinase reaction but may prove challenging to generalise to the genome scale. Current methods also often omit flux distributions from the input data, which has the caveat that reaction fluxes may be estimated to zero even in a non-equilibrium setting.

The model building approach presented in Adiamah *et al*. [7] showed that estimating kinetic parameters using metabolic and flux data could successfully reproduce experimental conditions under both steady-state and dynamic conditions. In an attempt to develop a solution addressing the combined challenges of building genome-scale integrative kinetic models, estimating kinetic parameters and measuring redundancy, we here present an approach to build a genome-scale kinetic model using generic equations, given a genome-scale flux distribution derived from FBA. We apply this approach to a genome-scale stoichiometric model of *Mycobacterium tuberculosis* metabolism developed by Beste *et al*. [15], which was shown to successfully simulate many of the growth properties of the bacterium. Tuberculosis (TB) remains one of the largest killer infectious diseases [16,17], and although significant advances were achieved in understanding the biology of *M. tuberculosis*, no new drug to treat tuberculosis has been developed in the last 30 years, making this organism an important subject for systems biology studies [18,19]. Our results show that an excellent agreement with flux values is obtained under several growth conditions, although kinetic parameters may vary in different conditions. Parameter variability analysis indicates that a high degree of redundancy remains present in model parameters when fluxes are the only constraining input.

## 2. Methods

### 2.1. Enzyme Kinetics and Rate Equations

We used the GRaPe software [7] to build a genome-scale kinetic model of *M. tuberculosis*. Rate equations for all reactions in the model are automatically generated by GRaPe based on the stoichiometry of the reaction. Reactions in the model assume a random-order mechanism as the sequential order of binding and releasing of substrates is often unknown. A key advantage of GRaPe over other tools is its ability to automatically generate rate equations for reactions, making it less error-prone and more time-efficient in building large-scale models. The King-Altman method [20] was used to derive rate equations based on the stoichiometry of a reaction and the enzyme mechanism.

The generic rate equations provided in [7] were used for all reactions of up to two substrates or products; these reactions can be of type uni-uni, uni-bi, bi-uni or bi-bi. For reactions of more than two substrates of products, the convenience kinetics was used [12]. The convenience kinetics equation, a generalised form of Michaelis-Menten kinetics, assumes a random-order mechanism and implements enzyme saturation and regulation. It can cover all possible reaction stoichiometries. For a reaction of type A1 + A2+ ... ↔ B1 + B2 + ..., the concentrations of substrates are represented by a vector *a* = (*a*_1_, *a*_2_, ...) and the concentrations of products are represented by a vector *b* = (*b*_1_, *b*_2_, ...). The flux *v*(*a*, *b*) is defined as:


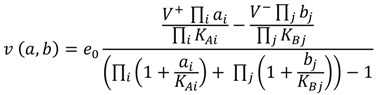
      (1) 

where *i* is the substrate index, j is the product index, *K_Ai_* represents the parameter (substrate constants) of the *i*^th^ substrate and *K_Bj_* that of the *j*^th^ product of the reaction, *e*_0_ is the concentration of enzyme, *V^+^* is the substrate turnover rate and *V^-^* is the product turnover rate.

### 2.2. Parameter Estimation

**Figure 1:**
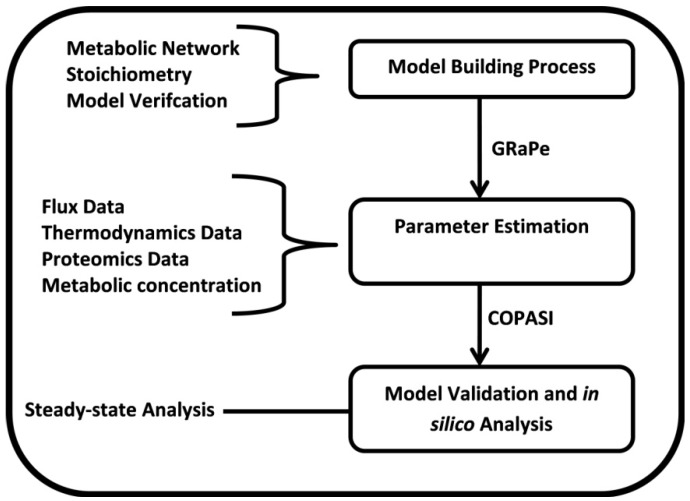
Schematic overview of the model development process.

Kinetic models have been shown to produce accurate and testable results [21], but due to the enormous number of kinetic parameters needed to define the system, the number of large-scale kinetic models remains relatively low. Furthermore, it was observed by Teusink *et al*. [22] that *in vitro* measurements of kinetic constants may not necessarily be representative of their numerical values *in vivo*. Currently, there are various software tools capable of performing parameter estimation: COPASI [23] provides several methods for parameter estimation including a genetic algorithm; SBML-PET [24] uses a stochastic ranking evolution strategy method to estimate parameters. However, flux constraints are excluded from the input data which can allow for a zero flux solution to be obtained even in non-equilibrium conditions. Since fluxes are not explicitly expressed as model elements, constraining parameters using those software is still not straightforward. Dynamic flux estimation shows that by verifying mass conservation in metabolic time-series data and integrating fluxes in the estimation of kinetic parameters values, the redundancy in model parameters can be reduced [25]. GRaPe uses a genetic algorithm to estimate kinetic parameters using flux values to constrain kinetic parameters. Figure 1 illustrates the process undertaken to reconstruct our kinetic model of *M. tuberculosis*. Other data sets can also be introduced into the parameter estimation process for constraining purposes, however the availability of comprehensive datasets on a large-scale is often lacking.

### 2.3. Parameter Variability Analysis (PVA)

One of the issues relating to parameter estimation is that of mathematical redundancy. The redundancy results in multiple sets of parameter values that can fit equally well to an experimental data set. A simple example of redundancy is when two parameters, *a* and *b*, are part of an equation in the form of *a* + *b* or *a* * *b*; if only their sum or product is known it is impossible to identify the value of *a* and *b* individually; if both the sum and product are known, then the value of *a* and *b* can be calculated. This example illustrates that the level of redundancy is dependent on the amount of experimental data used to constrain the estimation. When there is redundancy, the parameter values found in several runs of the estimation algorithm are likely to be different. In this article, we analysed the redundancy or ‘sloppiness’ in parameter estimation using parameter variability analysis (PVA). PVA allowed us to measure the range of changes in a set of parameter values when the estimation is repeated multiple times.

Once a model has been constructed or uploaded in GRaPe, PVA can be performed using the same data required to estimate parameter values for the model. The PVA algorithm works by repeating the estimation of kinetic parameters for the model multiple times using a genetic algorithm (GA). The GA works by populating a set of random initial parameter values; this is why results may differ after each run of the algorithm when there is redundancy. These estimated values are then optimised in an iterative manner until the maximum number of iterations is reached or a suitable solution is found. In GRaPe, GA uses flux and metabolic data to constrain parameters as illustrated in **Figure 1**. After each run of estimation, the objective function, which is a measure of the fit of the estimation to the original data, and kinetic parameter values are stored in a data file in a tabbed-delimited format. The results of PVA can then be exported to spreadsheet software and statistically analysed. The PVA function has now been fully integrated into the GRaPe software.

## 3. Results

### 3.1. The Genome-Scale Kinetic Model of *Mycobacterium Tuberculosis*

There are now several genome-scale metabolic reconstructions of *M. tuberculosis* [15,19,26] which can serve as a basis to construct a genome-scale kinetic model. In [15] the authors constructed a genome-scale metabolic network of *M. tuberculosis* using a reconstruction of *Streptomyces coelicolor* as a starting point. Genes were mapped between the two species using gene orthology clusters from the Kyoto Encyclopaedia of Genes and Genomes (KEGG) [27]. Using the KEGG and BioCyc databases and analysis of relevant research articles, the authors further supplemented the initial model. The final metabolic network of *M. tuberculosis* includes reactions needed for the synthesis of the cell membrane, complex lipids and carbohydrates, which are important for both growth and pathogenesis. Other metabolic pathways such as respiratory pathways and synthesis of biomolecules, which are specific to mycobacteria, were also modelled manually, as well as iron metabolism and transport reactions responsible for the import of carbon, nitrogen, minerals and compounds of high molecular weight. The final stoichiometric model reconstructed by the Beste group consists of 739 metabolites, 849 reactions and 726 genes; they calibrated their model by growing *Mycobacterium bovis* bacilli Calmette Guérin in a continuous culture and measuring parameters for steady-state growth. FBA was used to calculate substrate consumption rates. Their results showed a close agreement with experimentally determined values. The model was made available as a web-based interactive tool.

Using GRaPe [7], we created a genome-scale kinetic model of *M. tuberculosis* based on the stoichiometric model developed by Beste *et al*. [15]. GRaPe assigned an enzyme species to each reaction, which was then mapped to the corresponding gene(s) provided in the original reconstruction. All reactions were assumed to follow a random-order mechanism. We then used GRaPe to generate generic rate equations for all the reactions in the *M. tuberculosis* genome-scale network. The type of rate equation generated for each reaction was based on the stoichiometry of the reaction, as described in the Methods section. The resulting genome-scale model of *M. tuberculosis* contains 739 metabolites, 856 metabolic reactions and 856 enzyme species.

### 3.2. Parameter Estimation

We obtained flux distributions for three steady-states with glycerol being the only carbon source using the interactive web-based tool developed by Beste *et al*. [15]. The tool uses FBA to calculate flux distributions for the three steady-states with glycerol consumption at 0, 0.5 and 1 mmol/g dry weight (DW) respectively. It is worth noting that when glycerol uptake is set to zero the bacteria are still able to growth using glucose, albeit at a slow rate. For any given condition, the FBA solution is not unique as there are many alternative flux distributions that can sustain the same objective function, but only a particular solution is needed to provide a feasible flux distribution. Flux distribution data obtained under each experimental condition was then used as an input data source to estimate the parameters of our kinetic model. The precision of values in each dataset was limited to three decimal places for faster computing. A major difficulty in building genome-scale kinetic models is the lack of quantitative data available to fully define the model [21]; as a consequence, we set the initial concentrations of metabolites and enzyme species to an arbitrary unit of 1 by default.

We performed three separate parameter estimations for each of the three glycerol consumption rates. The kinetic parameters for each reaction in the model were estimated using GRaPe’s genetic algorithm. Model 1, with a glycerol consumption rate at 0 mmol/gDW/h, had 2297 kinetic parameters after parameter estimation; Model 2 had 2537 parameters with a glycerol consumption rate at 0.5 mmol/gDW/h, and Model 3 had 2931 parameters after parameter estimation with a glycerol consumption rate at 1 mmol/gDW/h. The difference in the number of parameters after each estimation was due to different numbers of reactions having a zero flux in each case. Furthermore, reactions with negative fluxes had their substrates and products swapped around to prevent having negative kinetic parameter values. The three models are provided in SBML format in Supplementary File 1, 2 and 3 respectively.

### 3.3. Model Validation

**Figure 2:**
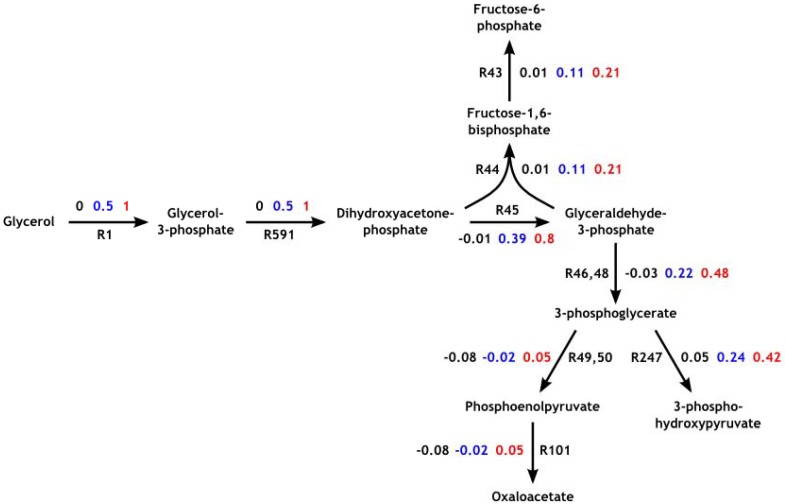
Main response of *Mycobacterium tuberculosis* to glycerol uptake rates at 0, 0.5 and 1.0 mmol/gDW/h. The network shows a selected set of reactions in the central metabolic pathways of *M. tuberculosis*. Reactions are represented using arrows and the positive direction of flux is indicated by the direction of the arrow (reaction reversibility is not represented). Numbers next to the arrows indicate flux values with black, blue and red colours corresponding to a glycerol uptake rate of 0, 0.5 and 1.0 mmol/gDW/h respectively; black numbers starting with R represent reaction numbers.

We performed a steady-state analysis for Model 1, 2 and 3 using COPASI. The results were then compared with the FBA flux distribution obtained from the Beste model under the same experimental conditions. Our verification analysis showed a near-perfect agreement between the results obtained from our models and the respective FBA simulation. Figure 2 shows the flux distributions in part of the central metabolic pathways; the complete comparisons of flux distributions for Model 1, 2 and 3 are provided in Supplementary File 4. These comparisons demonstrate the ability to accurately reproduce a steady-state flux distribution at the genome-scale using our model building approach.

**Figure 3:**
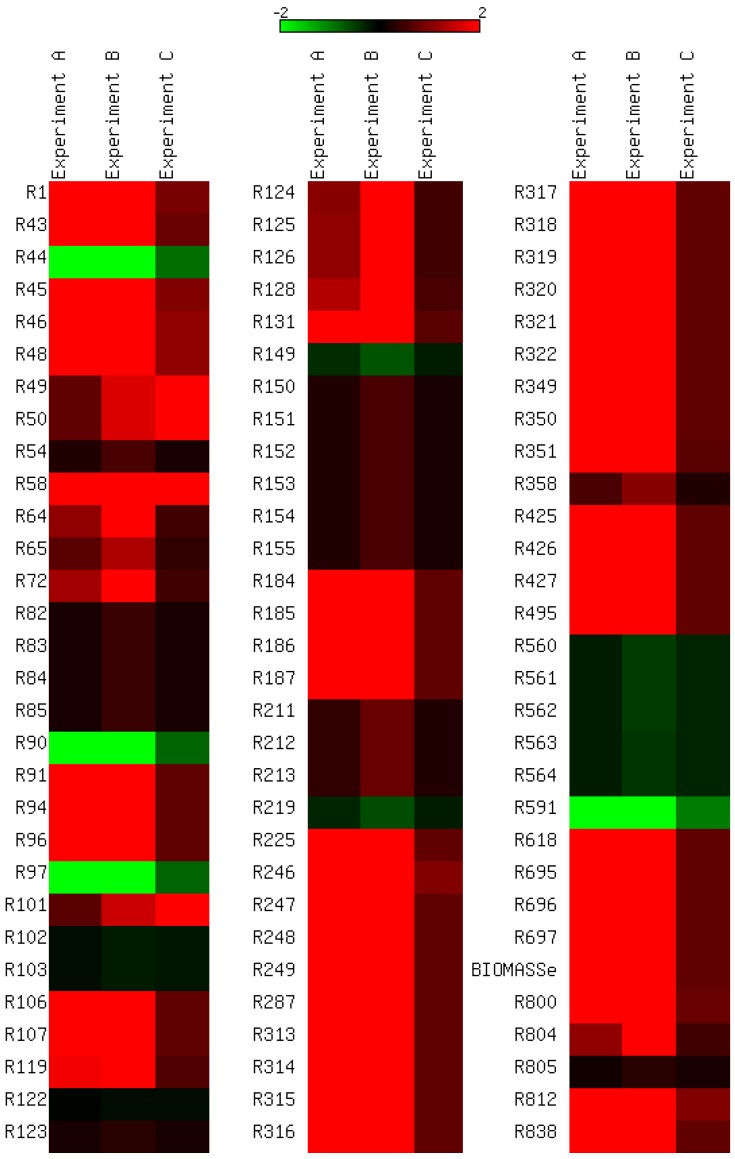
Relative changes in flux with changes in glycerol consumption rate. The response of selected reactions when the glycerol uptake rate is at 0 and 0.5 mmol/gDW/h is compared in Experiment A, between 0 and 1 mmol/gDW/h in Experiment B, and between 0.5 and 1 mmol/gDW/h in Experiment C. Only reactions with an absolute change greater than 10 % (0.1) and an absolute flux greater than 0.01 are shown. Green indicates a decrease and red indicates an increase in flux. Reactions identifiers are the same as in the Beste model.

We identified reactions that showed the greatest change in flux with respect to change in glycerol consumption rate (Figure 3). We calculated the relative change in fluxes between glycerol consumption rates at 0 and 0.5 mmol/gDW/h (Experiment A), 0 and 1 mmol/gDW/h (Experiment B), and 0.5 and 1 mmol/gDW/h (Experiment C). The most significant changes were observed in the enolase (R49) and pyruvate kinase (R50) reactions where a 100% increase in flux is observed when glycerol consumption was increased from 0 to 0.5 mmol/gDW/h and from 0.5 to 1 mmol/gDW/h.

### 3.4. Parameter Variability Analysis

To determine the degree of redundancy in the values of our estimated kinetic parameters, we performed PVA by repeating our estimation algorithm 100 times for Model 2. It is well known that different sets of parameters values can fit to experimental time-series data resulting in mathematical redundancy [28,29]. This means that running the parameter estimation 100 times may produce 100 different sets of parameter values that are able to fit the input data set equally well.

**Figure 4:**
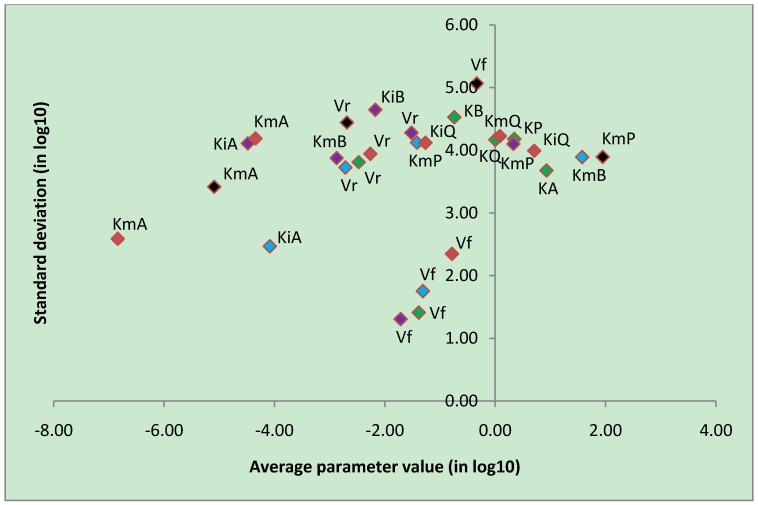
Average parameter values and standard deviations of estimated kinetic parameters after repeating the genetic algorithm 100 times. The parameters were classified into five reaction types: uni-uni (black), uni-bi (red), bi-uni (blue), bi-bi (purple) and convenience kinetics (green). Both axes of the graph are in logarithmic scale. *Vf* and *Vr* are the forward and backward reaction velocity, respectively; *KmA*, *KmB*, *KmP* and *KmQ* are Michaelis constants; *KiA*, *KiB*, *KiP* and *KiQ* are dissociation constants; *KA*, *KB*, *KP* and *KQ* are parameters of the convenience kinetics as defined in Equation (1).

In order to facilitate their interpretation, the results of PVA were split into five different categories based on the stoichiometry of the reaction (uni-uni, uni-bi, bi-uni, bi-bi and reactions of more than two substrates or products). The results of PVA show that many parameters are not strongly constrained (Figure 4). Separate graphs of average values and standard deviations for parameters under each reaction category are shown in Supplementary File 5. These results show that overall *V_f_*, the velocity of the forward reaction, is the most constrained parameter having the smallest standard deviation (Table 1). Since *V_f_* is directly related to the amount of enzyme and the expression level of the corresponding gene(s), it is expected to be more tightly linked to a particular condition, thus more constrained by a given flux distribution.

**Table 1:** Average parameter values and standard deviation (Stdev) for the most constrained parameters in logarithmic scale over 100 iterations of parameter variability analysis (PVA). Reactions are of type uni-uni, uni-bi, bi-uni, bi-bi, or convenience kinetics (CK).

Most constrained parameters			
Parameter	Reaction type	Average	Stdev
*V_f_*	CK	-0.78	2.35
*V_f_*	uni-bi	-1.39	1.41
*V_f_*	bi-uni	-1.71	1.31
*V_f_*	bi-bi	-1.31	1.75

The high degree of redundancy in the parameter values as indicated by PVA comes in support of our underlying assumption that accurate rate equations and kinetic parameters are not necessarily crucial in constraining the behaviour of the biological system. Nevertheless, the integration of genomic and proteomic data, together with metabolic and flux data, is expected to reduce mathematical redundancy as shown by previous studies [14,29].

**Figure 5:**
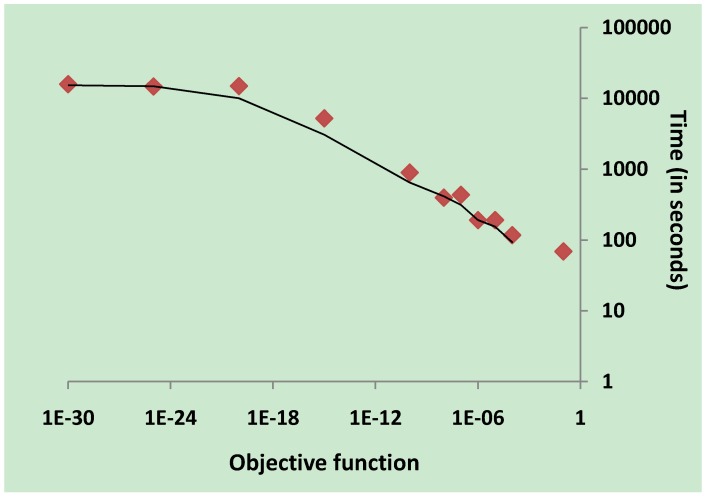
Computing times of parameter variability analysis (PVA) against changes in objective function. PVA was performed for a reaction with two substrates, two products, one enzyme and six kinetic parameters. For each PVA run, the summed squared mean distance measured between the simulated data and input data, known as the objective function, was set and the time taken to compute PVA results (running the genetic algorithm 100 times) was recorded. The results indicate a linear relation between the objective function and the computing time until the limits of computational precision are reached. Both axes of the graph are in logarithmic scale.

The computation of 100 sets of parameters for each reaction in Model 2 (with 739 metabolites, 856 metabolic reactions, 856 enzymes and 2537 kinetic parameters) for PVA took over 5 hours and 40 minutes. The relatively fast computing time was a result of reducing the objective function to 10^-4^ and limiting the data points to three decimal places in the input dataset. The objective function is the summed squared mean distance measured between the simulated data and input data. Reducing the objective function increased computing time but improves the quality of the parameter fit to input data. We performed an experiment to determine the relationship between the value of the objective function and the time taken to compute PVA for one reaction with two substrates, two products, one enzyme and six kinetic parameters (Figure 5). The results of this experiment indicate that the computing time for parameter estimation increases significantly when the objective function is reduced to 10^-10^ and beyond. The relationship that is observed between the objective function and computing time appears to be linear (PVA was computed on a desktop computer with a quad CPU having 3.00 GHz, 2.99 GHz processor speed and 4 GB of RAM).

**Figure 6:**
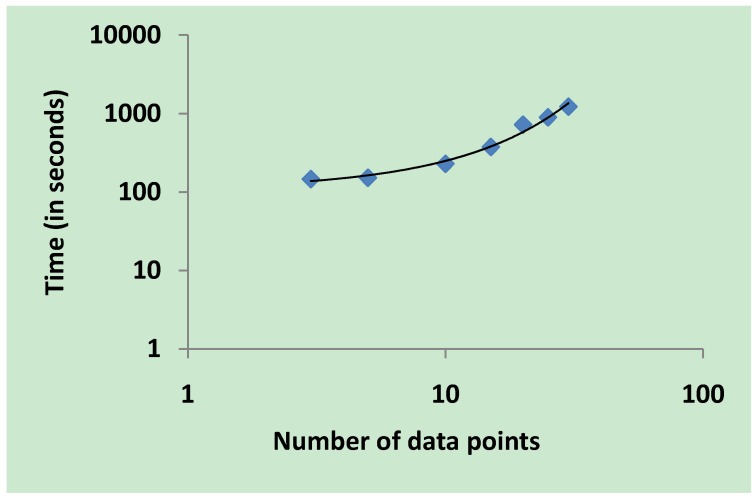
Relationship between number of input data points and computing time. PVA was performed for a single reaction of two substrates, two products, one enzyme and six kinetic parameters. PVA was repeated six times and for each iteration the number of data points in the input dataset for parameter estimation was increased from 3 to 30. The results show a rising curve in a non-linear shape.

Another variable that can increase computing time in parameter estimation is the number of data points in the experimental dataset. To examine how the number of data points influences computing time, we performed parameter estimation for a single reaction with two substrates, two products, one enzyme and six kinetic parameters (Figure 6). The result of this experiment indicates that the number of data points in the input dataset for parameter estimation increases the computing time in a non-linear manner. This explains why a relatively fast time of 5 hours and 40 minutes was recorded when PVA was performed for such a large model with 2537 kinetic parameters as the number of input data points was only three.

### 3.5. Validation on Model Integrity

We tested the predictive capability of our *M. tuberculosis* model and kinetic parameters by determining whether different conditions could be predicted without re-estimating kinetic parameters. To calibrate the model over a range of glycerol uptakes, we created a virtual time series containing ten repeats of each of the steady-state flux distributions obtained from input conditions with glycerol at 0, 0.5 and 1 mmol/gDW/h, respectively. The flux vector under each experiment was replicated ten times to create a data file with ten data points; we then merged the three individual files to create a data set with 30 time points, which was used as an input file for parameter estimation. Performing parameter estimation with this dataset took 125,022 seconds to complete with the objective function set at 10^-4^. The range of objective functions observed for individual reactions was between 10^-8^ and 10^‑20^. After parameter estimation, three steady-state analyses were performed with glycerol uptake at 0, 0.5 and 1 mmol/gDW/h using COPASI.

The resulting model was only able to predict the steady-state when glycerol update was at 0.5 mmol/gDW/h. Changing the glycerol level in this model resulted in simulation errors. A possible explanation for this unexpected observation is that combining three separate steady states is a fundamentally different experiment from having a dynamic change in glycerol level. Input flux distributions obtained from separate FBA simulations may be inappropriate to reproduce the dynamics of metabolic adjustment. To create a suitable training data set for dynamic modelling, intermediate data points covering the transition between steady states would be needed, but these data points cannot be obtained by FBA and require detailed experimental measurements. Another possible approach is that forward and backward reaction velocities (*V_f_* and *V_r_*), which can vary with different expression of the corresponding enzymes, should be allowed to vary in different conditions, whereas other parameters should remain the same. It is not currently possible to specify different levels of parameter constraints for different conditions in GRaPe, but this possibility may be added in the future.

## 4. Discussion

In this paper, we present the first genome-scale kinetic model of *Mycobacterium tuberculosis* based on generic kinetic equations. In recent years, there has been considerable progress in genome-scale data collection technologies, leading to ever increasing amounts of data in many organisms. However, the exploitation of such large datasets is proving challenging. For example, Ishii *et al*. [30] measured mRNA, protein and metabolite levels in multiple genetic and environmental perturbations in *E. coli*. Castrillo *et al*. [31] carried out comprehensive measurements at different growth rates in *S. cerevisiae*. Yus *et al*. [32] presented a global and multifaceted analysis of *Mycoplasma pneumoniae*. While each of these studies provided considerable new knowledge about the biology and cellular functions of their respective organism, a comprehensive model that is able to explain, and thus predict, such a large breadth of properties is still lacking for each of them. The main reason is that the construction of large kinetic models is arduous and challenging, and there are no established tools and techniques enabling the estimation of numerous kinetic parameters from large sets of heterogeneous data. Our aim is to show that, as an initial step, the construction of such genome-scale models given a comprehensive set of flux data can be achieved. The GRaPe tool assigns rate equations to all the reactions in the model based on the stoichiometry of the reaction. We successfully applied our methodology to the *M. tuberculosis* genome-scale metabolic network, resulting in a kinetic model with 739 metabolites, 856 metabolic reactions and 856 enzymes.

Predicting cellular behaviours *in silico* by examining the dynamics and properties of cellular processes has the potential to increase our understanding of biological systems. This makes it necessary to advance towards kinetic modelling in our drive to understand the detailed dynamics of cellular functions and their regulation. However, it is time-consuming and costly to experimentally measure all metabolite concentrations, reaction fluxes and kinetic parameters at the genome scale. Additionally, many kinetic equations are unknown and thus, standard rate laws have been used to describe metabolism. Liebermeister *et al*. [12], Adiamah *et al*. [7] and Ao *et al*. [10] have all shown that using generic rate equations, the dynamical behaviour of systems can be predicted without experimentally measuring all kinetic parameters. Constraint-based modelling fails in capturing the dynamics of cellular behaviour and is insufficient to provide insights into changes in metabolite concentrations.

Beste *et al*. [15] produced a constraint-based simulation of a genome-scale metabolic model of *M. tuberculosis* which was capable of predicting different growth conditions using FBA. The phenotype growth of 78% of mutant strains was correctly predicted by the Beste model. We built a genome-scale kinetic model of *M. tuberculosis* based on this stoichiometric model and showed that our model accurately reproduced genome-scale flux distributions under different growth conditions. The kinetic parameters used in our model were estimated using only flux values, therefore there remains a degree of redundancy in parameter values as illustrated by PVA. The results from PVA indicate that *V_f_*, the velocity of the forward reaction, is the most constrained parameter. The rest of the parameters in our model exhibit a high degree of redundancy. Banga [13] suggests that global optimisation methods are needed in an attempt to avoid finding local solutions. Additionally, there are suggestions indicating that due to the stochastic nature of biological systems, parameter estimation must account for this degree of stochasticity [33]. Reducing the value of the objective function in parameter estimation improves the quality of the kinetic parameters. However, we observed a significant increase in computing time when the objective function was reduced beyond 10^-8^. The compromise between computing time and more precise parameter values must always be considered when performing parameter estimation. Furthermore, our results also show that computing time increases non-linearly with the number of data points in the parameter estimation training data. When parameter estimation is being carried out for a system in steady-state, the number of data points can be reduced to lower the computing time.

An attempt was also made to constrain our kinetic parameters by training them with data based on three distinct experimental conditions. However, our model was able to predict only one state revealing the limits of using FBA steady states to constrain a dynamic model. Optimisation techniques can be used to estimate kinetic parameters based on simulated or experimental data [34,35]. However, these estimated parameter values are usually not unique given a set of an input data due to mathematical redundancy [29]. This redundancy means that multiple sets of parameter values can fit to an experimental data series equally well. There have been attempts in the past to reduce redundancy in parameter estimation. One noticeable approach is the use of Dynamic Flux Estimation (DFE) proposed by Goel *et al* [25] where there is a verification of mass conservation within metabolic time-series data and fluxes are expressed as functions of the relative variables affecting them. Although results from DFE show that redundancy can be reduced, the approach is computationally expensive due to the internal verification process.

## 4. Conclusions

In this article, we developed a genome-scale kinetic model of *Mycobacterium tuberculosis* based on generic kinetic equations. The model has 739 metabolites, 856 metabolic reactions and 856 enzymes. All kinetic parameters were estimated using a genetic algorithm based on the stoichiometry of reactions and flux distributions in the network. Our results show a near-perfect agreement with flux distributions under different growth conditions. The kinetic parameters used in our model were estimated using only fluxes, therefore there remains a degree of redundancy in parameter values. To further improve the predictive power of genome-scale dynamic models, the integration of more experimental data types including gene expression, proteomics and metabolomics, as well as the use of dynamic training data sets, will be needed. Nevertheless, our method for constructing a genome-scale kinetic model of *M. tuberculosis* represents a platform for further model development and analysis.

## Supplementary files

Supplementary File 1: Model of *M. tuberculosis* metabolism with parameters estimated for a glycerol consumption rate at 0 mmol/gDW/h (Model 1).

Supplementary File 2: Model of *M. tuberculosis* metabolism with parameters estimated for a glycerol consumption rate at 0.5 mmol/gDW/h (Model 2).

Supplementary File 3: Model of *M. tuberculosis* metabolism with parameters estimated for a glycerol consumption rate at 1 mmol/gDW/h (Model 3).

Supplementary File 4: Comparison between steady-state analyses of Model 1, 2, 3 and FBA flux distributions obtained from the Beste model under the same experimental conditions.

Supplementary File 5: Parameter variability analysis graphs of estimated kinetic parameters after repeating the genetic algorithm 100 times.

## Supplementary Files

Supplementary File 1 Supplementary (ZIP, 69 KB)

Supplementary File 2 Supplementary (ZIP, 74 KB)

Supplementary File 3 Supplementary (ZIP, 79 KB)

Supplementary File 4 Supplementary (XLSX, 103 KB)

Supplementary File 5 Supplementary (DOCX, 23 KB)
