# Supplementary material for: Construction of a Genome-Scale Kinetic Model of Mycobacterium Tuberculosis Using Generic Rate Equations
Source: Metabolites. 2012 Jul 3;2(3):382–97. doi: 10.3390/metabo2030382 (PMC3901218; doi:10.3390/metabo2030382)
Supplement: Supplementary File 5 — Supplementary (DOCX, 23 KB) [file metabolites-02-00382-s005.docx]

Parameter variability analysis graphs of estimated kinetic parameters after repeating the genetic algorithm 100 times.

# 1. Uni-uni reactions

# 2. Uni-bi reactions

# 3. Bi-uni reactions

# 4. Bi-bi reactions

# 5. Reactions with more than two substrates or products
